# Y Chromosome analysis of prehistoric human populations in the West Liao River Valley, Northeast China

**DOI:** 10.1186/1471-2148-13-216

**Published:** 2013-09-30

**Authors:** Yinqiu Cui, Hongjie Li, Chao Ning, Ye Zhang, Lu Chen, Xin Zhao, Erika Hagelberg, Hui Zhou

**Affiliations:** 1 College of Life Science, Jilin University, 130023 Changchun, People’s Republic of China; 2 Research Centre for Chinese Frontier Archaeology, Jilin University, 130012 Changchun, People’s Republic of China; 3 Institute of Archaeology, Chinese Academy of Social Sciences, 100008 Beijing, People’s Republic of China; 4 Department of Biosciences, University of Oslo, 0316 Oslo, Norway

## Abstract

**Background:**

The West Liao River valley in Northeast China is an ecologically diverse region, populated in prehistory by human populations with a wide range of cultures and modes of subsistence. To help understand the human evolutionary history of this region, we performed Y chromosome analyses on ancient human remains from archaeological sites ranging in age from 6500 to 2700 BP.

**Results:**

47 of the 70 individuals provided reproducible results. They were assigned into five different Y sub-haplogroups using diagnostic single nucleotide polymorphisms, namely N1 (xN1a, N1c), N1c, C/C3e, O3a (O3a3) and O3a3c. We also used 17 Y short tandem repeat loci in the non-recombining portion of the Y chromosome. There appears to be significant genetic differences between populations of the West Liao River valley and adjacent cultural complexes in the prehistoric period, and these prehistoric populations were shown to carry similar haplotypes as present-day Northeast Asians, but at markedly different frequencies.

**Conclusion:**

Our results suggest that the prehistoric cultural transitions were associated with immigration from the Yellow River valley and the northern steppe into the West Liao River valley. They reveal the temporal continuity of Y chromosome lineages in populations of the West Liao River valley over 5000 years, with a concurrent increase in lineage diversity caused by an influx of immigrants from other populations.

## Background

**Figure 1:**
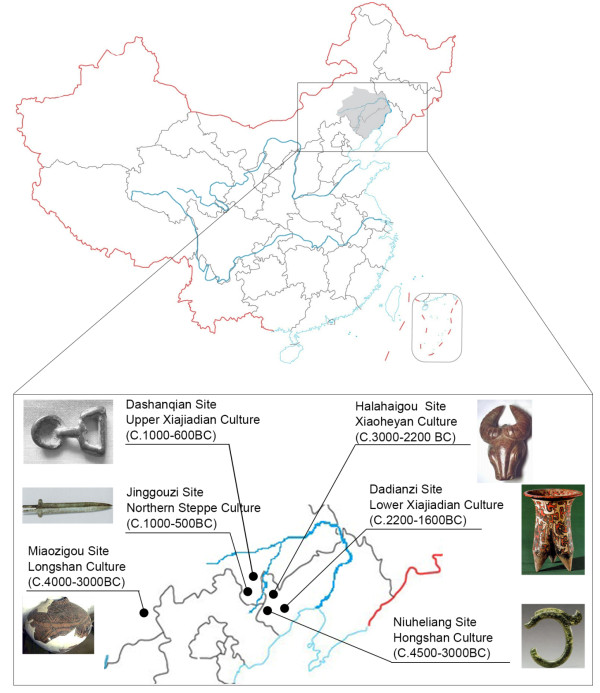
Geographic location of the archaeological sites in this study.

The West Liao River valley is situated in the southern region of Northeast China, between the Yellow River valley and the Eastern Eurasian steppe. It was occupied by a diverse sequence of human cultures that were different from both the farming populations of the Yellow River Valley and the nomads of the Eurasian steppe [1]. The sequence of cultures include the Hongshan culture (6500–5000 BP), Xiaoheyan culture (5000–4200 BP), Lower Xiajiadian culture (4200–3600 BP), and Upper Xiajiadian culture (3000–2700 BP) (Figure 1). The Hongshan culture is one of the most advanced Neolithic cultures in East Asia, with social stratification, distinctive painted pottery and elaborate jade ornaments. Archaeological investigations suggest that hunting- gathering was the main mode of subsistence, but they also indicate early use of cultigens in the Hongshan Culture. The Xiaoheyan culture adopted the basic features of the Hongshan culture, but had a simpler social organization. It was followed by the Lower Xiajiadian culture, which was marked by a gradual shift to agriculture and the establishment of permanent settlements with relatively high population densities, while retaining some of the hallmarks of the Hongshan culture [2]. It was replaced abruptly by a radically different culture, the Upper Xiajiadian, which was influenced by the Bronze Age cultures of the Northern China steppe [3]. The mode of subsistence shifted from hunting and early farming in the Hongshan and Xiaoheyan cultures, to advanced agriculture in the Lower Xiajiadian culture, and eventually to pastoral nomadism in the Upper Xiajiadian culture [4].

The West Liao River valley has a long history of human occupation since the Palaeolithic era. Because there is no natural barrier to the southwest or north, people have moved freely in this area since prehistoric times. However, the nature of these population movements and their contribution to any transition in subsistence strategy is a subject of heated discussion. A particularly interesting question is whether population replacement or gene flow accompanied the subsistence strategy transition process in the prehistoric West Liao River Valley [5].

Modern techniques of molecular analysis provide ideal tools to investigate whether changes in cultures and modes of subsistence were mediated by population replacement or cultural exchange. The Y chromosome, with its uniparental inheritance and low mutation rate [6], is used widely for tracing the history of human populations. Y chromosome analyses of present-day populations of Northeast Asia have revealed four principal Y chromosome haplogroups—C, D, N, and O—and indicate that extant patterns of genetic variation in East Asia were shaped to a large extent by a southern migration of humans in the Palaeolithic [7,8]. Ancient DNA analyses are particularly appropriate for investigating past migrations over extended time scales, as is the case for the West Liao River valley, where genetic data from present-day populations would lack sufficient discriminatory power. A previous study of Y chromosome variation in five ancient populations by the Yangtze River revealed considerable differences between haplogroup distributions in ancient and present-day populations. For example, haplotype O3d, found at high frequency in the ancient Daxi site in the middle reaches of the Yangtze River, is very rare in living people, except in the Hmong-Mien population of southern China and Southeast Asia. This suggests the ancient Daxi migrated south and became the ancestors of the present Hmong-Mien [9]. In another study, Y chromosome analysis of the ancient Kurgan people helped to unravel some of the history of early migrations in the Eurasian steppe and also provided new insights into the history of the south Siberian Kurgans [10]. A similar approach may help shed light on the patterns of prehistoric migrations in the West Liao River valley region.

In this study, prehistoric samples were collected from four archaeological sites representing the different time periods and cultures described above, and another two, the North nomad culture (Jinggouzi site) and the Yangshao Culture of the Yellow River valley (Miaozigou site) (Figure 1), were used as reference. We analyzed diagnostic single nucleotide polymorphisms (SNPs) in the non-recombining portion of the Y chromosome (NRY) of prehistoric samples (Table 1). A set of 17 Y short tandem repeats (STRs) were also analysed to confirm if ancient individuals of a particular haplogroup were related through the paternal line, even if buried in different tombs. They were also used to determine the detailed distribution of each haplogroup. By comparing our data with those from ancient and extant populations in the West Liao River valley and other surrounding regions in East Asia, we gained insight into the migration history and also evaluated genetic continuity in this region. These results will better help our understanding of the chief factors involved in the formation and transition of cultures in this region.

**Table 1:** Geographic locations and the Y-chromosome haplogroup distribution of prehistoric populations in this study

**Site**	**Culture**	**Time period**	**Age**	**Location**	**Sample size**	**N1(xN1a, N1c)**		**N1c**		**C/C3e**		**O3a (O3a3)**		**O3a3c**	
						**Number**	**Frequency**	**Number**	**Frequency**	**Number**	**Frequency**	**Number**	**Frequency**	**Number**	**Frequency**
Niuheliang	Hongshan Culture	Neolithic Age	6500-5000 BP	the border of Lingyuan County and Jianping County, Liaoning Province,China	6	4	66.7%	0	0	1(C)	13.7%	1	13.7%	0	0
Halahaigou	Xiaoheyan Culture	Neolithic Age	5000-4200 BP	Yuanbaoshan County,Chifeng city, Inner Mongolian Autonomous Region,China.	12	12	100.0%	0	0	0	0	0	0	0	0
Dadianzi	Lower Xiajiadian Culture	Early Bronze Age	4200-3600 BP	Aohan Banner,Chifeng city, Inner Mongolian Autonomous Region,China.	5	3	60.0%	0	0	0	0	2	40.0%	0	0
Dashanqian	Upper Xiajiadian culture	Late Bronze Age	3000-2700 BP	Harqin Banner,Chifeng city, Inner Mongolian Autonomous Region,China.	9	1	11.1%	3	33.3%	1(C3e)	11.1%	2	22.2%	2	22.2%
Jinggouzi	North nomad Culture	Late Bronze Age	3000-2500BP	Linxi County,Chifeng city, Inner Mongolian Autonomous Region,China.	12	0	0	0	0	12(C3e)		0	0	0	0
Miaozigou	Central Plain Culture	Neolithic Age	6000-5000BP	Chahar Right Front Banner, Ulanqab, Inner Mongolian Autonomous Region,China.	3	3	100.0%	0	0	0	0	0	0	0	0

Haplogroups were defined with the Y-SNP typing. Results are described in detail in Additional file 1**.**

## Results

### Authenticity of the ancient DNA results

Strict procedures were used to prevent modern DNA contamination, and we regard our results as authentic based on a number of different observations: a) The negative extraction and amplification controls were always free of contamination; b) the results were repeatable and reproducible, as verified by performing at least two duplicated extractions, and two duplicated amplifications of each extract; c) the Y-SNP and Y-STR profiles of the ancient individuals were different from those of the laboratory researchers; and d) we observed an inverse relationship between amplification efficiency and the size of the Y chromosome STRs.

### Y chromosome SNP analysis of ancient populations

**Figure 2:**
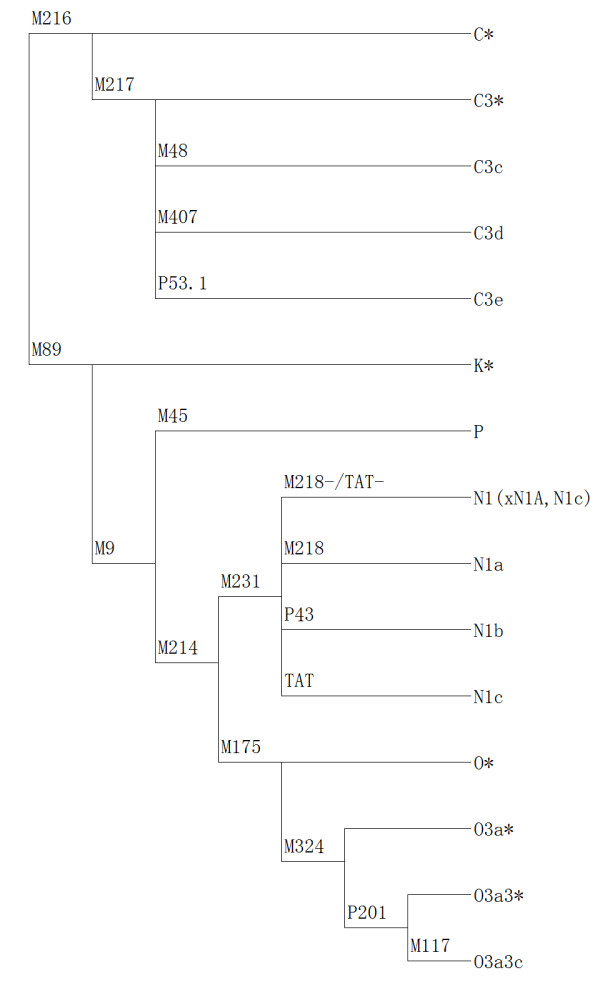
Phylogeny of Y-chromosomal haplogroups detected in this study.

The original sample consisted of 138 ancient individuals from six archaeological sites, of which 78 were classified as male using traditional morphological techniques. A further eight were found to be female by DNA typing of the sexually dimorphic amelogenin fragment. This left a total sample of 70 males, of which 23 either failed to amplify using Y chromosome SNP primers (21) or to yield consistent results (5). However, 47 of the 70 individuals, or over 60% of the samples tested, provided reproducible results. The 47 were then typed for a maximum of 18 Y chromosome SNPs (Figure 2), and could be classified into five different Y haplogroups (Additional file 1: Table S1). Some samples lacked sufficient DNA to permit further sub-classification.

The most ancient populations of the West Liao River valley exhibited a high frequency (71%) of haplogroup N1-M231. Because of the short amplicons needed for the ancient samples, it was not possible to type the diagnostic site P43 of sub-haplogroup N1b, so samples that yielded negative M128 and TAT mutations were defined as N1 (xN1A, N1c). Besides being the only haplogroup in the Halahaigou site, N1 (x N1a, N1c) was also predominant in the Niuheliang and Dadianzi sites. In the Dashanqian site, there were two subtypes of N1-M231: N1 (xN1a, N1c) and N1c-TAT. One of the nine Dashaqian samples was N1 (xN1a, N1c), and three were N1c (Table 1). N1 is particularly widespread in northern Eurasia, from the Far East to Eastern Europe. Its subtype, N1c, is found at low frequency but has high STR variability in northern China, suggesting that this region was N1c’s centre of expansion [11].

A single instance of O3a (xO3a3) was observed in the Neolithic Hongshan and Xiaoheyan sites, although this haplogroup was observed in just under half of the Bronze Age individuals. The Upper Xiajiadian individuals of the late Bronze Age had different subtypes of O3a-M324, O3a3c-M117. O3a-M324 is found today in most East Asian populations, and its subtype O3a3c-M117 occurs at the highest frequency in modern Sino-Tibetan populations [12,13].

C3-M217 is the most widespread haplogroup in Central Asia, South Asia, Southeast Asia, East Asia, Siberia and the Americas, but is absent in Oceania. Its sub-branch C3e-P53.1 is found only in Northeast Asia with low STR diversity, suggesting a recent origin in this region [6]. All individuals with the haplogroup C3-M217 in the ancient populations of the West Liao River valley belonged to the sub-branch C3e, except one from the Niuheliang site, who had an unidentified subtype. One instance of C3e-P53.1 was found in the Dashanqian site, while all 12 individuals of the Jinggouzi site belonged to this subtype. The Jinggouzi people originated in the North China steppe, and our findings support the view that C3e originated in the north.

### Y chromosome STR analysis

All ancient samples were analyzed at 17 Y chromosome STR loci. Due to DNA damage, only 21 of the 47 individuals yielded results for at least three loci in two independent extractions. Consensus data are reported in Additional file 1: Table S1. The DYS389II, DYS438 and DYS635 loci frequently failed to amplify, probably because of their longer length. The inverse relationship between amplification efficiency and PCR fragment is further support for the authenticity of the extracted DNA, as ancient DNA is presumably degraded while modern DNA contamination would exhibit longer fragment lengths.

There are only two Y-chromosome haplotypes in the Jinggouzi site suggesting that individuals are paternally closely related, despite being buried in separate tombs. In the other sites in our study, we detected no potential paternal relatives among ancient individuals of the same haplogroup.

## Discussion

### Y chromosome characteristics of the prehistoric population

The West Liao River valley was a cradle of Chinese civilization, together with the valleys of the Yellow River and Yangtze River, and there is considerable interest among scholars in the origin and expansions of the ancestors of the present-day inhabitants. Extensive analyses of extant populations have revealed that the most common Y chromosome haplogroup today is O-M175 (58.8%, n=176), followed by C3-M217(23.8%), N-M231(8.5%), and several relatively rare haplogroups, namely D-M174, Q1a1-M120, and R-M207 [8,14]. Our data reveal that the main paternal lineage in the prehistoric populations was N1 (xN1a, N1c), present in about 63% of our combined sample of all cultural complexes. It was the predominant haplogroup in the Neolithic period (89%), and declined gradually over time (Table 1). Today it is only found at low frequency in northeast Asia (Table 2). There appears to be significant genetic differences between ancient and extant populations of the West Liao River valley (P<0.001).

**Table 2:** Frequency distribution of ancient Y-chromosome haplogroups in the extant populations of East Asia and North Asia

**Geographical region**	**Population**	**Size**	**other**	N*	**N1 (×N1c,N1a)**	**N1c**	C*	**C3e**	O*	**O3a (×O3a3c)**	**O3a3c**	**Literature**
West Liao-River valley												
	Prehistoric Population	32	0	0	50.0	9.4	3.1	3.1	0	15.6	6.3	This study
South East Asia												
	Buyi	35	5.7	5.7	0	0	0	0	60.0	17.1	11.4	Xue YL,2006
	Li	34	0	0	0	0	0	0	97.1	0	2.9	Xue YL,2006
	She	7	0	0	0	0	0	0	85.7	0	14.2	Sengupta S,2006
	She	34	0	0	0	0	2.9	一	44.4	29.4	23.5	Xue YL,2006
	She	51	0	0	0	0	0	0	94.1	0	5.9	Hammer F,2006
	Hani	34	1	0	11.8	0	17.6	一	52.9	0	14.7	Xue YL,2006
	Yao (Bama)	35	5.7	2.9	0	0	17.1	一	42.9	34.3	0	Xue YL,2006
	Yao (Liannan)	35	0	0	0	0	2.9	一	25.7	51.4	20.0	Xue YL,2006
	Qiang	33	33.3	0	0	0	0	0	39.4	6.1	21.2	Xue YL,2006
	Yi	43	25.6	0	30.2	0	2.3	一	16.3	0	27.9	Hammer F,2006
	Han (Chengdu)	34	0	0	2.9	0	11.8	一	55.9	0	29.4	Xue YL,2006
North East Asia												
	Han (Harbin)	35	2.9	0	2.9	2.9	14.3	一	40.0	0	37.1	Xue YL,2006
	Han (Yili)	32	25.0	0	0	0	6.3	一	53.1	6.3	9.4	Xue YL,2006
	Han	44	13.6	2.3	6.8	0	4.5	一	31.8	0	40.9	Hammer F,2006
	Ewenki	26	7.7	0	0	0	57.7	一	15.4	0	19.2	Xue YL,2006
	Ewenki	57	66.7	一	一	一	31.6	1.8	一	一	一	Zhong H,2010
	Hezhe (Heilongjiang)	45	2.2	0	17.8	0	28.9	一	31.1	0	20.0	Xue YL,2006
	Manchu (Liaonign)	52	9.6	1.9	3.8	0	26.9	一	42.3	0	15.4	Hammer F,2006
	Manchu	35	5.7	5.7	8.6	0	25.7	一	34.3	0	20.0	Xue YL,2006
	Mongolian	149	14.1	0	6.0	2.0	52.3	一	9.4	0	16.1	Hammer F,2006
	Mongolian (innermongolia)	22	22.7	4.5	一	一	50.0	4.5	18.2	一	一	Zhong H,2010
	Mongolian (innermongolia)	45	8.9	0	0	13.3	46.7	一	13.3	0	17.8	Xue YL,2006
	Mongolian	65	23.1	0	3.1	7.7	53.8	一	6.2	0	6.2	Xue YL,2006
	Uygur (Urumqi)	31	77.4	0	9.7	0	3.2	一	3.2	0	6.4	Xue YL,2006
	Uygur (Yili)	39	66.7	0	25.6	5.1	10.3	一	5.1	0	10.3	Xue YL,2006
	Korea (Yanji)	25	12.0	0	4.0	0	12.0	一	56.0	0	16.0	Xue YL,2006
	Xibe (Xinjiang)	41	19.5	2.4	9.8	4.9	26.8	一	19.5	0	17.1	Xue YL,2006
	Xibe (Xinjiang)	61	6.6	18.0	一	一	26.2	9.8	39.3	一	一	Zhong H,2010
	Oroqen	31	3.2	0	6.5	0	61.3	一	16.1	6.5	6.5	Xue YL,2006
	Oroqen	7	0	0	0	28.6	71.4	一	0	0	0	Sengupta S,2006
	Oroqen	22	0	0	0	4.5	90.9	一	4.5	0	0	Hammer F,2006
	Japan	47	59.6	38.3	4.3	0	10.6	一	2.1	0	21.3	Xue YL,2006
	Japan	23	78.3	0	0	0	13.0	一	0	0	2	Sengupta S,2006
	Dawoer	39	7.7	0	0	7.7	30.8	一	46.2	0	7.7	Xue YL,2006
	Hui (Ningxia)	62	37.1	8.1	一	一	17.7	1.6	35.5	一	一	Zhong H,2010
	Hui	35	48.6	0	0	0	22.9	一	22.9	0	5.7	Xue YL,2006
	Tibetan	35	51.4	0	8.6	0	0	0	0	0	40.0	Xue YL,2006
	Tibetan	105	59.0	0	2.9	0	1.9	一	2.9	0	34.3	Hammer F,2006
North Asia												
	Tuvan	55	45.5	0	10.9	27.3	14.5	0	1.8	0	0	Pakendorf B_2006
	Yakut	184	3.3	0	0.5	94.0	2.2	0	0	0	0	Pakendorf B_2006
	Evenk	40	2.5	0	27.5	0	70.0	0	0	0	0	Pakendorf B_2006

Note:

1. South East Asia and North East Asia populations are defined by the geographic separation of Yangtze River.

2. 一 indicates that no data exists for this population.

3. The * refer to all the other sub-haplogroups not used in this study.

Previous analyses showed that there were different frequency distributions of the sub-haplogroups used in this study in both ancient and extant populations of adjacent regions. The Yellow River valley, located in the southwest region of the West Liao River valley, was one original centre of agriculture in China. O3-M122 is the most abundant haplogroup in both ancient (80%, n=5) and extant population (53%, n=304) of the region [8,13], but the frequency of O3-M122 only began to rise in the West Liao River valley in the Bronze Age. The ancient West Liao River valley population is significantly different from both the ancient Yellow River Valley population (P<0.01), and the extant Yellow River Valley population (P<0.01). The Miaozigou site, about 500 km west of the West Liao River valley in the central/southern region of Inner Mongolia, was settled by people of the northern branch of the Yangshao culture, an important Neolithic farming culture along the Yellow River. Our analysis of three ancient Miaozigou individuals revealed that they all belong to haplogroup N1(xN1a, N1c), while the main lineage of the Yellow River valley culture is O3-M122 [9]. The existence of N1(xN1a, N1c) in the Miaozigou site could be evidence for the expansion of the Hongshan culture during its heyday, a view supported by archaeological evidence of Hongshan influences at the Miaozigou site [15]. However, the small sample size of our current ancient genetic material and the lack of data for earlier time periods means an alternate explanation [16], in which N1(xN1a, N1c) existed across the region prior to the Neolithic, is still a possibility.

The main haplogroups of Northern steppe nomad population were C3 (50.7% in the Mongolian, n=285) [8,17,18], and N1c (94% in the Yakut, n=184) [19]. The ancient individuals from the Jinggouzi site, a Northern Steppe nomadic culture on the western fringes of the West Liao River valley, carry a single haplogroup, C3e, divided into two sub-types on the basis of Y chromosome STR analysis. Previous mtDNA data have shown that the Jinggouzi people have closely related mtDNA types [20], suggesting that the Jinggouzi site was settled by family groups migrating from the northern steppe within a short period, which is in agreement with archaeological results [21]. Therefore, the prehistoric people of the West Liao River valley carried the characteristic N1 (x N1a, N1c) lineage, and appear both culturally and genetically distinct.

### Prehistoric migrations in relation to cultural transitions

The Lower Xiajiadian culture (LXC) was an early Bronze Age culture with a highly developed agricultural society, with a subsistence strategy quite different from the hunting-gathering strategy typical of the Hongshan culture. However, the LXC people retained the microliths (tips of hunting weapons) and custom of dragon worship typical of the Hongshan culture. Most archaeologists agree that during the transition from the Neolithic to the Bronze Age, migrants carried farming technology from the Yellow River valley to surrounding areas including the West Liao River valley. In the Dadianzi people of the LXC, O3a is the main haplogroup after N1(xN1a, N1c). The former was previously shown to be the characteristic lineage for ancient populations along the Yellow River and Yangtze River valleys [9]. Previous mitochondrial DNA analyses of the Dadianzi population showed that the LXC people probably included immigrants from the Central Plains [22]. The archaeological analyses showed that farming tools and ceramic techniques can be traced to cultures from the Yellow River Basin [3]. Both the ancient genetic and archaeological data suggest that immigrants from the Yellow River valley, of type O3a, may have migrated into the West Liao River valley and influenced changes to the existing culture, but genetic drift cannot be ruled out as the cause for the observed frequencies.

The Upper Xiajiadian culture (UXC) of the late Bronze Age succeeded the LXC but was completely different from the LXC. The UXC people mainly practiced animal husbandry and made bronze objects decorated with animal and other natural motifs in the style of the Eurasian steppes. The UXC individuals of the Dashaqian site had higher Y chromosome haplogroup diversity, with a lower frequency of the LXC lineage. Only one individual carried N1 (×N1a, N1c), the prevalent haplogroup before the UXC period. The O3-M122 type could have been inherited from LXC, but the existence of two different sub-types of O3, O3a (xO3a3) and O3a3c, implies continuous northward gene flow from the Yellow River valley. It is worth noting that the two northern haplogroups N1c and C3e first appeared in the ancient peoples of the Dashaqian site. N1c-TAT has the greatest frequency in populations from Northern Eurasia (see Table 2), and 94% of Yakuts belong to this haplogroup [19]. 33.3% of Dashaqian samples were N1c, and the present-day distribution of the ancient haplotype based on one STR profile search is mainly Northern Asia. The presence of N1c in the UXC might suggest that there is immigration from the north Eurasian steppes during this period.

The Jinggouzi site is situated northwest of the West Liao River Valley, and was occupied by northern nomadic tribes during similar time periods (3000-2500BP) as the Dashanqian site. All ancient samples of the Jinggouzi site were assigned to C3e, suggesting northern nomads might have entered the West Liao River valley from the northwest. C3e is rare in modern populations, and is only found in Northeast Asia.

Because the farming LXC was replaced by the nomadic UXC and no transitional type has yet been found, it had been suggested that there might have been large-scale immigration or even population replacement by northern Asian nomads [23]. Y chromosome data show immigration components from both northern steppe tribes and farmers from the Yellow River valley. However, because all original LXC lineages in this investigation were retained in the UXC gene pool, we tend to believe that while immigrant nomads from the north played an important part in the cultural transitions in this region, they probably did not replace the preceding populations in the West Liao River valley. Instead, the cultural transitions were more likely the result of adaptations to a new lifestyle caused by climate change.

### Temporal continuity of paternal lineages in the West Liao River valley

The origin and development of the prehistoric populations of the West Liao River valley, a cross road of continuous migration events, is expected to involve complex processes and population admixture. Our prehistoric population data show that the principal lineages in the region remained relatively constant from the Neolithic to the Bronze Age. In the historic period, the region was controlled mainly by nomads, including the Nüzhen, Mongolians and Manchu. The genetic structure of this period can be deduced from data of Xibe, an extant minority in Xinjiang, from the northwestern region of China. The Xinjiang Xibe originated in Northeast China and were sent to Xinjiang in 1764 by the Qing emperor to defend the frontier [24]. This population carries the original Y chromosome lineages of the prehistoric population of the West Liao River valley, with a high frequency of C3e (Table 2), whose genetic structure is similar to that of the Upper Xiajiadian.

In modern times, especially the last century, a massive number of immigrants from the south poured into this region. To investigate the extent of continuity in the paternal lineages, we examined the present-day patterns of distribution of the Y chromosome lineages observed in our ancient populations (Table 2). Except for O3a, the lineages of the prehistoric people are present today at low frequencies in the West Liao River valley. O3a continued to enter the West Liao River valley during the expansion of the Yellow River valley culture, displacing or replacing the original lineages. Today, N1 (xN1a, N1c) and C3e are mostly found in the northern Han and the northeast minority populations such as the Mongolians, Manchu, Oroqen, Xibe and Hezhe, although at low frequencies. Yi is the only population which has a relatively high frequency of N1 (xN1a,xN1c) in southern China. According to the archaeological record, one of the original branches of the ancestral Yi population was the Diqian, a nomadic ethnic group who lived in the northern steppes from 5000 to 3000 BP [25], which may explain the origin of N1(xN1a,xN1c) in the Yi people.

## Conclusion

Our data demonstrate the temporal continuity of Y chromosome lineages in the populations of the West Liao River valley during the past 5000 years, with a concurrent increase in lineage diversity but at markedly different frequencies caused by the influx of immigrants from other populations. During the cultural transitions occurring in this region, the immigration had an effect on the genetic structure of populations in this region, but no population replacement was found. More ancient data from adjacent geographic regions and time periods, especially from the Yellow River Valley and Eastern Eurasian steppe, will be needed to more accurately describe the contribution of migration to cultural transition in this region.

## Methods

### Archaeological sites and samples

The geographic locations of the archaeological sites in this study have an average temperature of ca. 10°C, with a cold, dry climate excellent for the preservation of organic remains. The bone samples in this study were from six different archaeological sites: Niuheliang, Halahaigou, Dadianzi, Dashanqian, Jinggouzi, and Miaozigou. The first four are highly representative of the prehistory of the West Liao River valley, while the last two were used as references (Figure 1 and Table 1). The Jinggouzi was a typical Northern nomadic culture, and the Miaozigou represents the Yangshao Culture of the Yellow River valley. A number of different tombs were sampled for each cultural complex. Two teeth for each individual were collected for DNA analysis. Sex identification was performed using morphological and molecular methods as described previously [26].

### Methods to avoid DNA contamination and monitor authenticity

Appropriate precautions were taken to ensure the authenticity of the ancient DNA results. All pre-polymerase chain reaction (PCR) steps were performed in a positive pressure laboratory dedicated to ancient DNA located in the Research Center for Chinese Frontier Archaeology of Jilin University. Different rooms were used for sample preparation, DNA extraction, and setting up PCR. Post-PCR procedures were carried out in a different building. Surfaces were cleaned regularly with a 10% sodium hypochlorite solution and UV light (254 nm), and full-body protective clothing, facemasks and gloves were worn. Gloves were changed frequently. All consumables were purchased as DNA-free and additionally sterilized by autoclaving at 121°C for 15 min, while reagents were irradiated with UV light for at least 20 min. Every PCR assay included extraction and amplification controls. To check for reproducibility, the experiments were performed in parallel using duplicate teeth of each individual in the Molecular forensic lab in the College of Life Science of Jilin University. At least two PCR amplifications per SNP were done in each laboratory. To identify potential contamination from laboratory personnel, the mtDNA and STR profiles of all staff in the project were obtained. All pre-PCR steps were carried out by women, minimizing the risk of contamination by male DNA.

### DNA extraction

Teeth were soaked in a 10% sodium hypochlorite solution for 20 min, and washed with ultra-pure water and 100% alcohol. Each tooth was then exposed to UV light for 30 min on each side. The teeth were ground to fine powder in a 6750 Freezer Mill (Spex SamplePrep, USA), refrigerated with liquid nitrogen, and stored at −20°C. For each extraction, a quantity of ground tooth (ca. 0.2 g) was incubated for 24h at 50°C with shaking (220 rpm/min), in 4 ml of an incubation buffer consisting of 0.5M EDTA, 0.5% SDS and 3 mg/ml proteinase K. Afterwards, the DNA was purified using the QIAquick PCR Purification Kit (Qiagen, Germany), according to the manufacturer’s protocol. A short fragment of the D-loop region in the mitochondrial genome (nt16052-16142) was amplified for each sample. Only the samples which had positive PCR product would be tested further for nuclear genetic material.

DNA extracts were quantified by real time PCR, using an ABI Prism 5700 Sequence Detection System (Applied Biosystems, USA) and the Quantifiler® Human DNA Quantification Kit (Applied Biosystems, USA) according to the manufacturer’s protocol.

### Y chromosome STR and SNP analysis

Eighteen biallelic markers (Figure 2) that characterize the most prevalent lineages in Eastern Asia were tested using a hierarchical genotyping strategy [27]. First, the six Y chromosome markers C-M216, F-M89, K-M9, P-M45, NO-M214 and N-M231 were genotyped. Afterwards, the C-M216-derived individuals were subjected to further typing of four biallelic markers, C3-M217, C3c-M48, C3d-M407, and C3e-P53.1, which define four sub-haplogroups. The NO-M214-derived individuals were further typed at eight biallelic markers, N1a-M128, N1b-P43, N1c-TAT, O-M175, O3-M122, O3a-M324, O3a3-p201, and O3a3c-M117, which define six sub-haplogroups. Primers for PCR amplification were designed using Primer Premier 5.0, and primer sequences are shown in Table 3. PCR amplifications were done as described by Karafet et al. (2008), but increasing the number of cycles to 40, using previously described primers [28,29]. The length of the PCR amplicons was typically between 100 and 200 base pairs (bp). Together with the published data [6,8,17,18,22,30], the frequencies of ancient haplogroups in extant Asia populations are summarized in Table 2. The significance analysis of difference in haplogroup frequency was performed using Fisher’s exact test for the ancient populations due to small sample size, otherwise we use the chi square test.

**Table 3:** Information for the 18 Y-SNP markers and primer sequences

**Marker**	**Amplicon size (bp)**	**PCR primer sequnces (5’→3’)**		**Test method**
		**Forward**	**Reverse**	
C/M216	109bp	TCACTTTTATATCCTCAACCA	AATCTGAATTCTGACACTGC	Sequence
C3/M217	101bp	ACTTGTGAAGGAGAATGAAAA	GCATTTGATAAAGCTGCTGTG	Sequence
C3c/M48	140bp	AAACAATATGTATGCTAATTTTGCT	TCAATGTAAATGTTAGTATAAGGATG	Sequence
C3d/M407	114bp	TCTTACTGAAAGTTGGGGAC	GATAATCGCTTGTCTCTTGG	Sequence
C3e/P53.1	123bp/121bp	AAACCCTGGAGAGGACCAACGA	ACACTATGAACCAATCCCACCC	Sequence
F/M89	125bp	CCACAGAAGGATGCTGCTCA	CACACTTGGGTCCAGGATCAC	Sequence
K/M9	128bp	GGACCCTGAAATACAGAAC	AAGCGCTACCTTACTTACAT	Sequence
P/M45	129bp	GGGTGTGGACTTTACGAAC	AAATCCTACTATCTCCTGGC	Sequence
NO /M214	119bp	MACTGGAAAGAAAAAGAATGCTG	ATGGAAATGCCACTTCACTC	Sequence
N/M231	113bp	CCTGGAAAATGTGGGCTC	TTCTTTGACGATCTTTCCCC	Sequence
N1a/M128	123bp/121bp	ATCTACCTCTTTCAAACTGT	GAACTGCCTCTTATAAAATCAT	Sequence
N1b/P43	108bp	ACGGAGTCTCGCTCTGTCG	GCTACTTGGGAGGCTGAGG	Sequence
N1c/Tat	115bp	GAGAAGGTGCCGTAAAAGTG	GGACTCTGAGTGTAGACTTGTG	Sequence
O/M175	110bp/105bp	TCAACTCAACTCCAGTGATTTA	TAATGATACCTTTTTTTCTACTGA	PAGE
O3/M122	120bp	CAGATACTGTGACTTTGAG	GAAATGAATAAATCAAGGT	Sequence
O3a/M324	120bp	GGAACATAGCAAGACCCAAAAT	CAAATTGATTTCCAGGGATACAT	Sequence
O3a3/P201	114BP	FTAAGCAATGAAGGTAGAAGG	ATTTAGAATAATATTTACTGAGCA	Sequence
O3a3c/M117	117bp/113bp	AAAATAACTCACCAAAGGAAT	AGATGATAGAAAAACATAATA	PAGE

PAGE: Polyacrylamide gel electrophoresis.

Y chromosome STR of the ancient samples was performed on 17 loci (DYS19, DYS385, DYS389I, DYS389II, DYS390, DYS391, DYS392, DYS393, DYS437, DYS447, DYS438, DYS439, DYS448, DYS456, DYS458, DYS635 and Y GATA H4) using the AGCU^®^ mini STR Kit (AGCU ScienTech, China). Experimental conditions were as recommended by the manufacturer, but the number of PCR cycles was increased to 40. STR products were analyzed on an ABI Prism 310 Genetic Analyzer with GeneMapper software 4.2. The STR haplotypes were examined to identify potential paternal relationships, and each STR profile was cross-checked with the Y chromosome Haplotype Reference Database (YHRD) (http://www.yhrd.org).

## Availability of supporting data

The data set supporting the results of this article is included within the article and its additional file.

## Competing interests

The authors declare no competing interests.

## Supplementary Material

Additional file 1: Table S1 Detailed sample information, Y-SNP and Y-STR genotype data. Click here for file
